# Repurposing of Anthocyanin Biosynthesis for Plant Transformation and Genome Editing

**DOI:** 10.3389/fgeed.2020.607982

**Published:** 2020-12-03

**Authors:** Yubing He, Min Zhu, Junhua Wu, Lejun Ouyang, Rongchen Wang, Hui Sun, Lang Yan, Lihao Wang, Meilian Xu, Huadong Zhan, Yunde Zhao

**Affiliations:** ^1^State Key Laboratory of Crop Genetics and Germplasm Enhancement, Nanjing Agricultural University, Nanjing, China; ^2^National Key Laboratory of Crop Genetic Improvement and National Center of Plant Gene Research (Wuhan), Huazhong Agricultural University, Wuhan, China; ^3^Guangdong Laboratory for Lingnan Modern Agricultural Science and Technology, Guangdong University of Petrochemical Technology, Maoming, China; ^4^Key Laboratory of Plant Resource Conservation and Sustainable Utilization, South China Botanical Garden, Chinese Academy of Sciences, Guangzhou, China; ^5^Section of Cell and Developmental Biology, University of California, San Diego, La Jolla, CA, United States

**Keywords:** CRISPR, transgene-free, anthocyanin, rice, AAC

## Abstract

CRISPR/Cas9 gene editing technology has been very effective in editing genes in many plant species including rice. Here we further improve the current CRISPR/Cas9 gene editing technology in both efficiency and time needed for isolation of transgene-free and target gene-edited plants. We coupled the CRISPR/Cas9 cassette with a unit that activates anthocyanin biosynthesis, providing a visible marker for detecting the presence of transgenes. The anthocyanin-marker assisted CRISPR (AAC) technology enables us to identify transgenic events even at calli stage, to select transformants with elevated *Cas9* expression, and to identify transgene-free plants in the field. We used the AAC technology to edit *LAZY1* and *G1* and successfully generated many transgene-free and target gene-edited plants at T1 generation. The AAC technology greatly reduced the labor, time, and costs needed for editing target genes in rice.

## Introduction

CRISPR/Cas9 genome editing technology has been widely used to generate targeted modifications of genes in many plant species (Feng et al., 2013; Li et al., 2013a; Mao et al., 2013; Miao et al., 2013; Nekrasov et al., 2013; Shan et al., 2013; Xie and Yang, 2013; Gao et al., 2015, 2016). Gene editing efficiency correlates with the expression level of *Cas9* and higher expression of *Cas9* usually leads to an increase in editing efficiency. Selecting transgenic plants with elevated *Cas9* expression by analyzing Cas9 protein concentrations is laborious and time-consuming, Therefore, *Cas9* expression levels are often monitored indirectly. Coupling a fluorescence marker with the *Cas9* expression unit provides an effective approach for identify plants with elevated Cas9 concentrations (Gao et al., 2016; Wang and Chen, 2020). Another creative approach was to couple *Cas9* expression with a guide RNA that can lead to a visible phenotype when the target gene is edited. Genes involved in trichome development have been targeted to visibly monitor gene editing efficiency (Wang et al., 2015; Miki et al., 2018). Whereas, the aforementioned methods have been effective, fluorescence markers need special equipment and are not suitable for field conditions. Targeting trichomes is very useful for Arabidopsis, but may not be effective for monocots such as rice. Additional markers that can be indicative of the levels of transgene expression will be very useful in conducting gene editing experiments.

In addition to improving gene editing efficiency, a main emphasis in editing plants/crops is to generate edited plants without any transgene residuals. Crops with any CRISPR/Cas9 component residual will unlikely receive approval for commercial applications from government regulatory agencies. The continuous presence of gene editing elements in plants may cause genetic instability and off-target events. Several strategies have been reported for isolating transgene-free and target gene-edited plants. Transient expression of *Cas9* and gRNA genome editing complex through Agrobacterium-mediated infiltration has led to the identification of target gene-edited plants without any transgene residues in tobacco (Chen et al., 2018). Cas9 and gRNA can also be assembled into ribonucleoprotein (RNP) complexes and then delivered into plant cells using nano particles (Doyle et al., 2019; Landry and Mitter, 2019) or bombardment (Svitashev et al., 2016; Zhang et al., 2016). Because RNP contains no DNA, any edited plants are considered transgene-free, However, RNP method is extremely inefficient because the majority of the regenerated plants are not transformed due to a lack of selection pressure. Identification of transgene-free, target gene-edited plants can also be assisted by fluorescence markers (Gao et al., 2016; He et al., 2017a; Yu and Zhao, 2019; Ouyang et al., 2020) and positive or negative selection against chemicals (Lu et al., 2017; Wu et al., 2019; Li et al., 2020), Moreover, we developed a CRISPR/Cas9 gene editing system in which the transgenes undergo automatic self-elimination after the target gene has been edited (transgene killer CRISPR (TKC), greatly improving the efficiency for isolating the desired plants (He et al., 2018; 2019). Whereas, TKC system reduces labor and time needed for conducting gene editing in rice, it does not have a proxy for indicating *Cas9* expression levels.

Anthocyanins are a large class of secondary metabolites (Tanaka et al., 2008), which are widely distributed in various tissues including flowers, stems, leaves, and fruits. Anthocyanins are water-soluble polyphenol pigments with vivid red, blue, purple, and other colors, Therefore, anthocyanins potentially can be used as a visible marker for visualizing transgenic events or the presence of transgenes, However, anthocyanin biosynthetic pathway is very complex and consists of at least eight genes (Zhu et al., 2017; Zheng et al., 2019). It is not realistic to couple the entire anthocyanin biosynthetic pathway with Cas9/gRNA units in a plasmid because the resulting plasmid would be too big for efficient transformation or cloning. Fortunately, anthocyanin biosynthesis pathway is under the control of key transcription factors including MYB, bHLH, and WD40 family genes (Zhang et al., 2014; Liu et al., 2015; Xu et al., 2015). Almost all of the anthocyanin biosynthesis structural genes are activated (or enhanced) by the MYB–bHLH–WD40 (MBW) complex (Xu et al., 2015; Zhu et al., 2017; Zheng et al., 2019), and the MYB protein is believed to be the key component in the allocation of specific gene expression patterns (Jaakola, 2013; Xu et al., 2015). Based on the number of imperfect repeats (R) domain(s), MYB genes are divided into four major groups: 1R (R1/2, R3-MYB), 2R (R2R3-MYB), 3R (R1R2R3-MYB), and 4R (four R1/R2-like repeats) (Liu et al., 2015). The R2R3-MYB is an activator for the synthesis of anthocyanins (Borevitz et al., 2000; Liu et al., 2015).

The *ZmC1* gene, encoding an R2R3 MYB, regulates the synthesis of anthocyanins in the corn aleurone layer (Cone et al., 1986; Paz-Ares et al., 1986). Its ortholog, *OsC1* gene controls the color change in leaf sheath and stigma in rice. Ectopic expression of *OsC1* in different rice varieties results in accumulation of anthocyanins in various tissues and organs (Gao et al., 2011; Chin et al., 2016; Zhao et al., 2016), Therefore, ectopic expression of *OsC1* may be used to activate anthocyanin biosynthesis, thus providing a visible marker in rice. Here, we show that the expression of *OsC1* under the control of the rice *ACTIN* promoter leads to the accumulation of anthocyanin in calli, young seedlings, leaf vascular tissue, and grains, providing a visible marker for selecting transgenic plants. When the *OsC1* unit is coupled with CRISPR/Cas9 units, target genes in almost all of the anthocyanin positive plants have been edited, suggesting that the threshold of visible anthocyanin accumulation is a good indication of high level *Cas9* expression, Moreover, anthocyanin accumulation allows a visual differentiation between transgenic and non-transgenic plants at T1 generation without the need of conducting PCR and other molecular analysis, greatly accelerating the isolation of transgene free and target-gene edited plants.

## Results

### Activation of Anthocyanin Biosynthesis Serves as a Marker for the Presence of Transgenes

**Figure 1:**
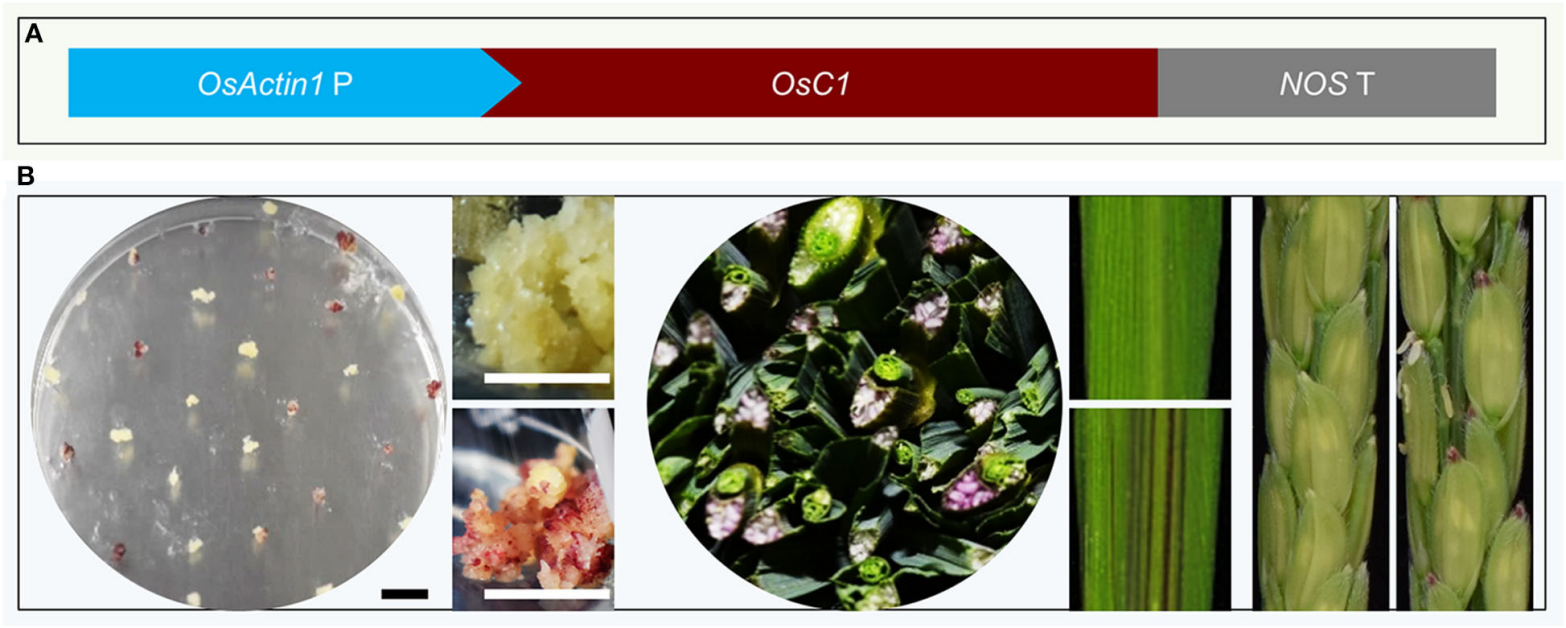
The *pACTIN-OsC1* activates anthocyanin biosynthesis in rice. **(A)** A schematic description of the *pACTIN-OsC1* unit in the vector used for rice transformation. The anthocyanin regulation gene *OsC1* is under the control of the rice *OsACTIN1* promoter. *NOS* T refers to the terminator of the nopaline synthase gene from *Agrobacterium tumefaciens*. **(B)** The *AAC* plasmid induced obvious accumulation of anthocyanin (purple color) in rice calli, vascular tissue in stems (transverse section), leaves, and the tip of grains. Scale bars 1 cm in tissue culture images.

Ectopic expression of the *R2R3-MYB* gene in rice and other plants activated anthocyanin biosynthesis (Borevitz et al., 2000; Shin et al., 2006; Han et al., 2009; Li et al., 2017; Zhang et al., 2019). We chose the *OsC1* (GenBank ID: MK636605), an *R2R3-MYB* gene, from the rice cultivar Heishuai (Zheng et al., 2019), which displays obvious purple color in leaves, stems, and grains. We placed *OsC1* cDNA under the control of the rice *ACTIN1* promoter, a constitutively activated promoter (Figure 1A). When plasmids containing the *pACTIN-OsC1* unit were transformed into rice, we observed that anthocyanin accumulated in calli, leaves, and the tip of grains (Figure 1B), demonstrating that the *pACTIN-OsC1* unit can be used to produce a visible color, which can facilitate the identification of transgenic events in calli and transgenic plants.

### Coupling Anthocyanin Biosynthesis With Genome Editing Units

**Figure 2:**
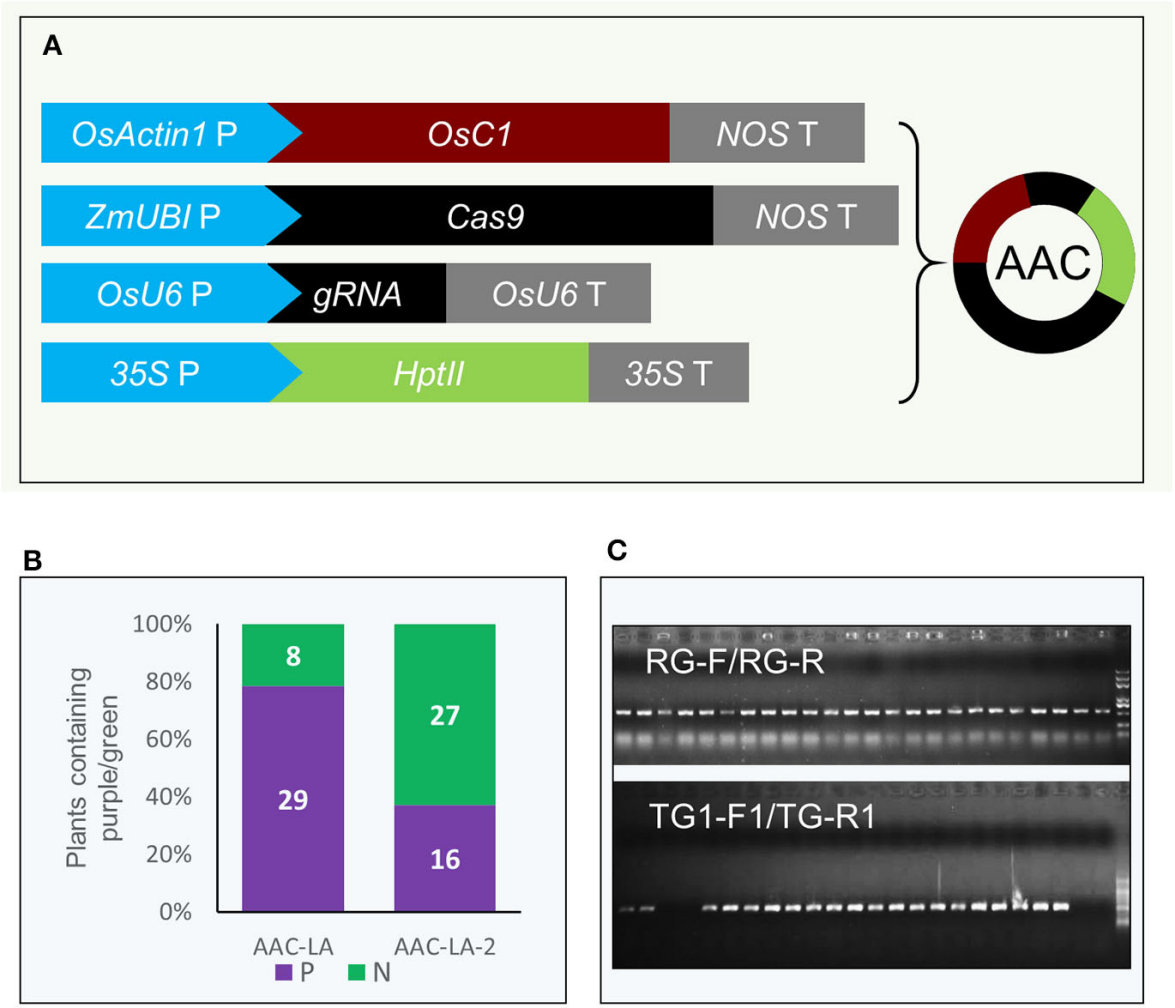
Anthocyanin accumulation as a marker for the presence of transgenes in gene editing experiments. **(A)** Coupling the *pACTIN-OsC1* unit with the CRISPR/Cas9 genome editing units in the *AAC* (Anthocyanin-marker Assisted CRISPR) plasmid. The anthocyanin regulatory gene *OsC1* is under the control of the rice *OsACTIN1* promoter. *NOS* T refers to the terminator of the nopaline synthase gene from *Agrobacterium tumefaciens*. The rice codon-optimized *Cas9* was placed under the control of the maize *UBIQUITIN* promoter. *OsU6* P and *OsU6* T represent rice *U6* promoter and terminator, respectively. gRNA refers to guide RNA. The transcription of *hygromycin phosphotransferase II* gene *HptII* is under the control of the *CaMV 35S* promoter and the transcription was terminated with a *CaMV 35S* terminator. **(B)** Not all antibiotic resistant plants generated purple color in T0. AAC-LA was transgenic event screening from the plate with two rounds hygromycin selection (10 days in a round). AAC-LA-2 was regenerated from the calli on normal plate after 7 days hygromycin selection (only one round). “P” and “N” represent purple and normal color plants, respectively. **(C)** Verification of the transgenes in the rice plants by PCR. “RG-F/RG-R” and “TG-F1/TG-R1” refer to the primer pairs used for detection the presence of rice genomic DNA and the T-DNA of *AAC* plasmid, respectively. The primer information was described in Supplementary Table 3.

We placed the *pACTIN-OsC1* unit in adjacent to the cassettes for *Cas9* expression and gRNA production, resulting in the *AAC* plasmid (*A*nthocyanin-marker *A*ssisted *C*RISPR) (Figure 2A). We first tested whether our *AAC* plasmids can achieve efficient editing of the *LAZY1* gene, which leads to agravitropic growth when compromised (Li et al., 2007), providing a visible phenotype for gene editing events. We cloned the *LAZY1* gRNA unit into the *AAC* plasmid to generate the *AAC-LA* plasmid, which was subsequently transformed into rice through *Agrobacterium*-mediated transformation. We found that almost 80% of the T0 plants regenerated from the plate with two rounds hygromycin selection (10 days in a round) had purple color tissues (Figure 2B). Furthermore, we noticed that transferring the calli to antibiotic-free media after 7 days hygromycin selection (only one round), resulted in about 40% regenerated plants with purple color tissues (Figure 2B), suggesting that activation of anthocyanin can be used for selecting transgenic events with a decreased usage of antibiotic. Whereas, antibiotic or herbicide resistant markers are potentially detrimental to the environment, the native anthocyanin biosynthesis pathway offers a good alternative for selecting transformants.

We observed that all of the T0 plants that displayed purple color contained the *OsC1* expression cassette revealed by our PCR analyses (Figure 2C). We also found that some of the hygromycin-resistant plants without the purple color also contained the transgene, suggesting that a threshold level expression of the *OsC1* is required in order to produce a visible color. The results suggest that we might be able to use the accumulation of anthocyanin as a proxy for monitoring Cas9 expression levels.

### Facilitating the Isolation of Transgene-Free and Target Gene-Edited T1 Plants Using the Anthocyanin-Marker

**Figure 3:**
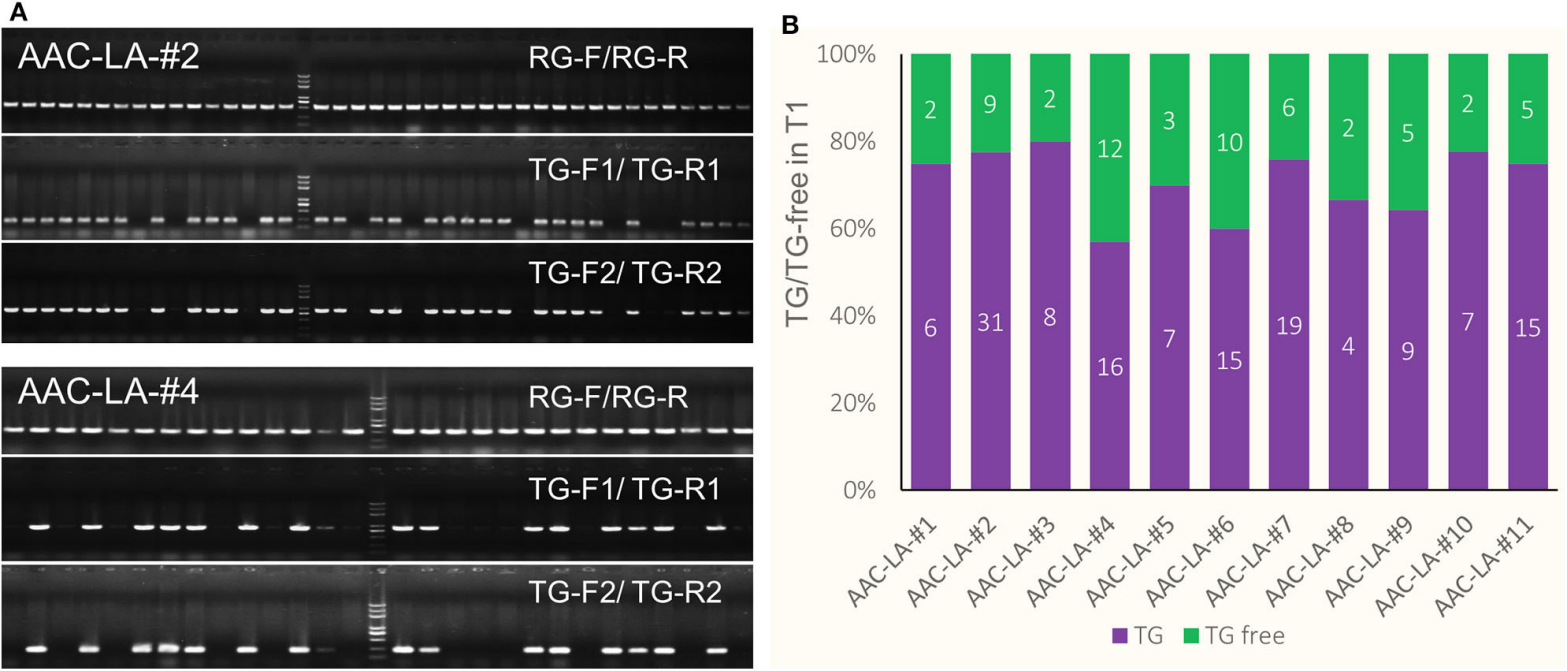
Isolation of transgene-free T1 plants by anthocyanin-marker assisted strategy. **(A)** The transgene-free plants from the progeny of the T0 plants that had purple color were identified by PCR and visual screen. AAC-LA–#2 and AAC-LA-#4 represent two AAC-LA T0 plants with purple color, respectively. **(B)** The segregation of the transgene (TG) and transgene-free (TG-free) plants from 11 AAC-LA T0 lines. The numbers on purple and green columns represent the numbers of purple and normal colored plants at T1 generation. The results were consistent with the results identified by PCR. “RG-F/RG-R” refers to primer pair used for checking the quality of rice genome DNA. “TG-F1/TG-R1,” and “TG-F2/TG-R2” represent two primer pairs used for detection the presence of T-DNA, respectively. The primers information was described in Supplementary Table 3.

We hypothesized that we might be able to visually identify transgene free T1 plants generated from the T0 plants that had obvious anthocyanin accumulation. We also used PCR-based assays to further confirm the absence of transgenes. We used two pairs of primers, TG-F1/TG-R1 and TG-F2/TG-R2 to amply specific DNA fragments from the *AAC* plasmid (Figure 3A). We found that the PCR results matched perfectly with the color based visual screen. For example, among 40 and 28 T1 plants from the AAC-LA-#2 and AAC-LA-#4 T0 plants, 9 and 12 plants did not display obvious anthocyanin accumulation, respectively (Figure 3B). Our PCR results identified the same plants as transgene-free (Figure 3A). Further analyses of the T1 plants from the AAC-LA–#1 to # 11 T0 plants demonstrated that the transgene-free T1 plants identified on basis of anthocyanin accumulation matched with those transgene-free plants identified by PCR assays (Figure 3B). Our results demonstrated that the anthocyanin-based visual screen for transgene-free T1 plants was effective and accurate. Such a visual assay greatly reduced labor and time for identifying transgene-free plants.

### Molecular Characterization of the Transgene-Free and Gene-Edited Plants

**Figure 4:**
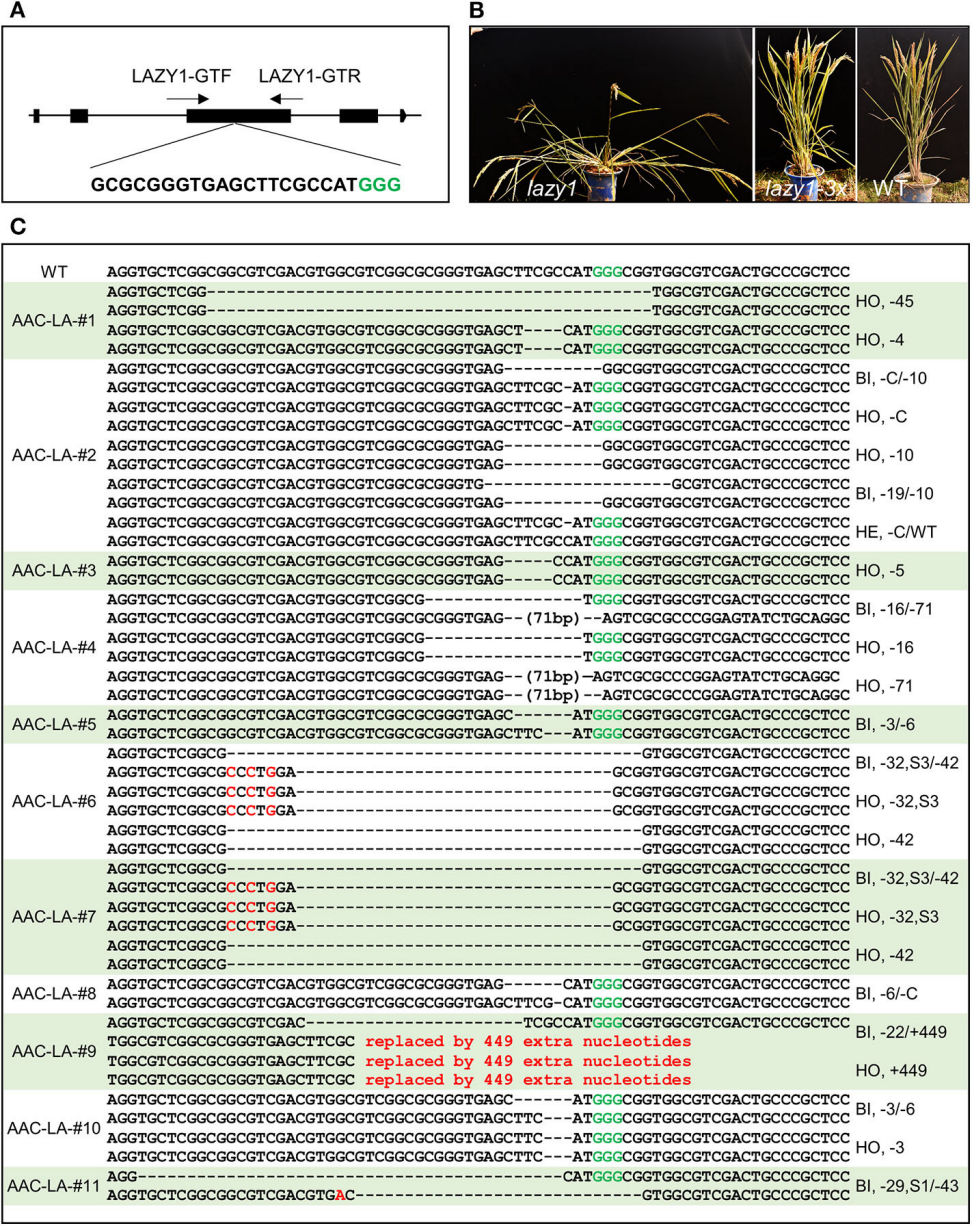
Identification of transgene-free and target gene-edited *lazy1* mutants. **(A)** Target site of *LAZY1* used in *AAC-LA*. A target sequence in the *LAZY1* gene includes the PAM site GGG (marked green) is shown. LAZY1-GTF and LAZY1-GTR were the genotyping primer pair of *LAZY1* CRISPR plants. Primers were listed in Supplementary Table 3. **(B)** Loss-of-function *lazy1* mutants displayed an obvious tiller angle phenotype. WT, *lazy1* and *lazy1-3x* refer to wild type, loss-of-function *lazy1* mutant, and in-frame (multiple of three base pairs deletion) *lazy1* mutant plants, respectively. **(C)** The mutation forms of the transgene-free and CRISPR-edited *lazy1* mutants. The PAM site “GGG” in green required for Cas9 cleavage is marked in green. “WT” refers to the wild type plants. “HO,” “HE,” and “BI” represent homozygous, heterozygous, and bi-allelic genotypes, respectively. “-” refers to a deletion of one base pair. “S3” and “S1” means three and one base pair(s) substitution, respectively.

To analyze the molecular lesions in the *LAZY1* gene in the T1 plants generated from the AAC-LA T0 plants that had displayed anthothyanin accumulation, we directly sequenced the *LAZY1* fragment amplified using the primers of LAZY1-GTF/ LAZY1-GTR (Figure 4A). We analyzed T1 plants from 11 independent T0 plants, which had obvious anthocyanin accumulation (Table 1, Supplementary Table 1). Every single plant contained mutations at the target site except that a few plants failed to generate quality sequencing data. The T1 plants analyzed were either homozygous or bi-allelic except one T1 plant from AAC-LA-#2, which was heterozygous, demonstrating the power of AAC technology in gene editing (Table 1, Supplementary Table 1). For comparison, our previously experiments using our regular CRISPR vectors usually had the editing efficiency <80% (He et al., 2017b).

**Table 1:** Genotypes and segregation patterns in the T1 plants generated from the AAC-LA.

**Plants No**.	**Genotypes**	**Segregation ratio**	**Transgene-free ratio**
AAC-LA–#1	HO,−45	6/8	1/6
	HO,−4	2/8	1/2
AAC-LA–#2	BI,–C/−10	18/40	3/18
	HO,–C	12/40	2/12
	HO,−10	7/40	2/7
	BI,−40/−10	1/40	0/1
	BI,−19/−10	1/40	1/1
	HE,–C/WT	1/40	1/1
AAC-LA–#3	HO,−411	4/9	0/4
	HO,−5	5/9	1/5
AAC-LA–#4	BI,−16/−71	15/26	5/15
	HO,−16	3/26	1/3
	HO,−71	8/26	5/8
AAC-LA–#5	BI,−3/−6	3/7	1/3
	HO,−3	2/7	0/2
	HO,−6	2/7	0/2
AAC-LA–#6	BI,−32,S3/−42	11/23	4/11
	HO,−32,S3	7/23	3/7
	HO,−42	5/23	2/5
AAC-LA–#7	BI,−32,S3/−42	13/25	5/13
	HO,−32,S3	5/25	1/5
	HO,−42	7/25	1/7
AAC-LA–#8	BI,−6/–C	3/5	1/3
	HO,−6	1/5	0/1
	HO,–C	1/5	0/1
AAC-LA–#9	BI,−22/+449	5/13	1/5
	HO,−22	2/13	0/2
	HO, +449	6/13	2/6
AAC-LA–#10	BI,−3/−6	4/8	1/4
	HO,−3	3/8	1/3
	HO,−6	1/8	0/1
AAC-LA–#11	BI,−29,S1/−43	11/18	4/11
	HO,−29,S1	6/18	0/6
	HO,−43	1/18	0/1

“*HO,” “HE,” and “BI” represent homozygous, heterozygous, and bi-allelic genotype, respectively. The numbers in the column of “Genotypes” refer to the numbers of base pair changes in each line. The details of the mutations of each genotype were shown in Supplementary Table 1. “Segregation ratio” refers the ratio of the number of the plants shown the genotype in the column of “Genotypes” to the total number of the plants in the T1 line*.

Further analysis of the mutations revealed some interesting patterns. We observed four *lazy* alleles in the progeny of AAC-LA−2 T0 plant: a “C” deletion, a 10-base pair deletion, a 40-base pair deletion, and a 19-base pair deletion. The *lazy* alleles resulted in six combinations of genotypes: eighteen “–C/−10” biallelic mutants, twelve “–C” homozygous mutants, seven “-10” homozygous mutants, one “−40/−19” biallelic, one “−19/−10” biallelic mutant, and a wild type plant in the T1 plants (Table 1, Supplementary Table 1). The first three genotypes were predominant, accounting for 92.5% (37/40) of the T1 plants. Because of the complex genotype (likely mosaic) in T0 plants, it is extremely important to analyzed T1 plants that no longer have the *Cas9* transgenes.

Using anthocyanin as a visible marker for transgenes (Figure 3) of the T1 plants, we easily identified transgene-free *lazy1* mutants of at the T1 generation from multiple independent T0 plants (Figures 4B,C). Some of T0 plants did not show the *lazy* mutant phenotype, but they harbored mutations with 3x of base pair deletion, such as the lines from AAC-LA–#1, 5, 6, 7, 8, 10 (Figure 4C, Table 1, Supplementary Table 1). Such 3x mutations did not cause frameshift and likely produced functional protein.

### Testing the AAC Gene Editing System With Another Target

**Figure 5:**
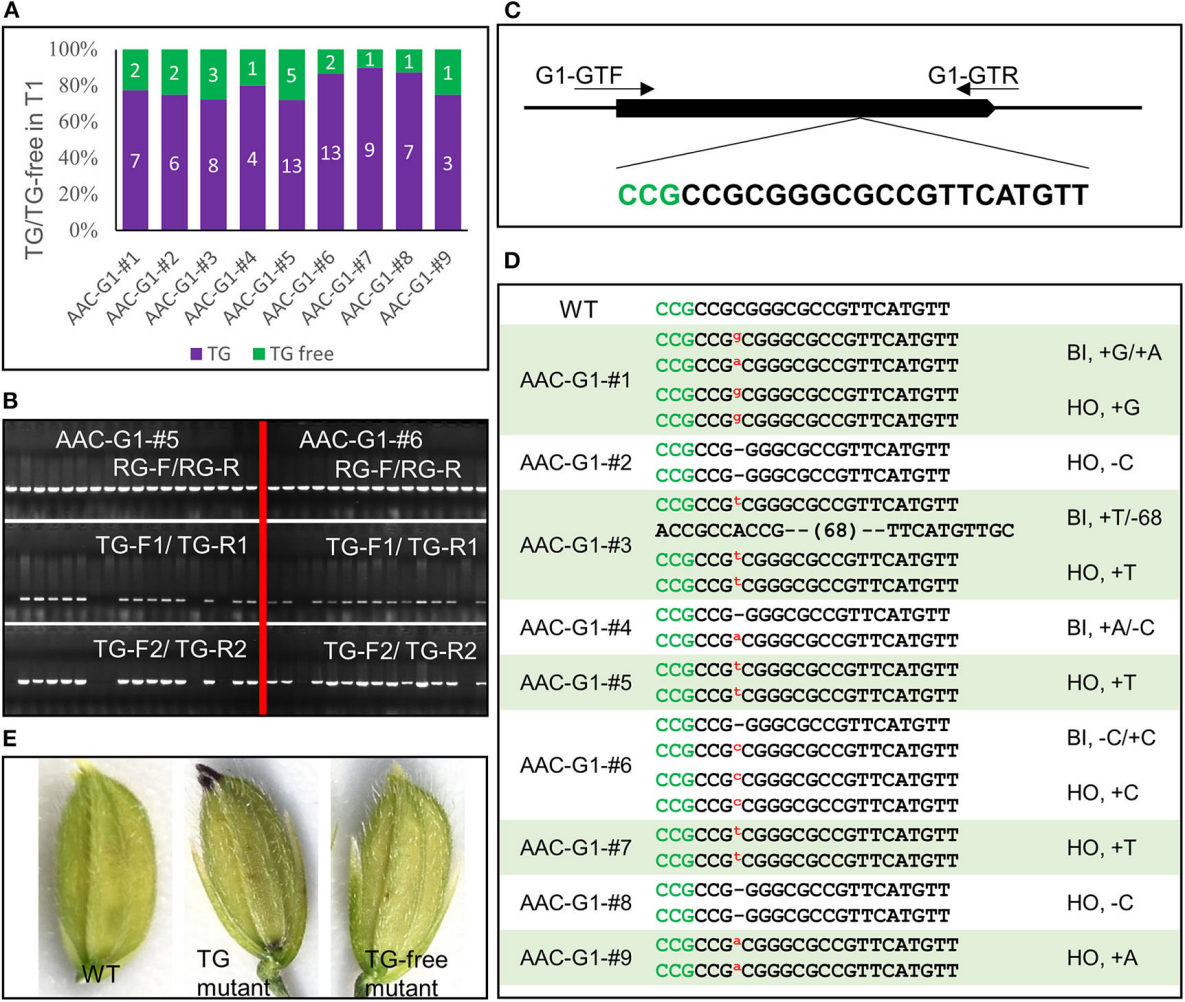
Identification transgene-free and gene-edited *g1* mutants. **(A)** The segregation of the transgene (TG) and transgene-free (TG-free) plants from 9 AAC-G1 T1 lines. The numbers on purple and green columns represent the numbers of purple and normal color plants in T1 lines which were consistent with the results identified by PCR, respectively. **(B)** The transgene-free plants from the progeny of the purple-colored T0 plants were identified by PCR. AAC-G1–#5 and AAC-G1-# 6 refer to two AAC-G1 T0 plants containing the purple color, respectively. “RG-F/RG-R” refers to primer pair used for checking the quality of rice genome DNA. “TG-F1/TG-R1” and “TG-F2/TG-R2” represent two primer pairs used for detection the presence of T-DNA, respectively. The primers information was described in Supplementary Table 3. **(C)** Target site of *G1* used in *AAC-G1* plasmid. A target sequence including the PAM site CCG (marked green) was chosen. G1-GTF and G1-GTR are the genotyping primer pair of *G1* CRISPR plants. Primers were listed in Supplementary Table 3. **(D)** The mutation forms of the transgene-free and CRISPR-edited *g1* mutants. The PAM site “CCG” required for Cas9 cleavage is marked in green. “WT” refers to the wild-type plants. “HO” and “BI” represent homozygous and bi-allelic genotypes, respectively. “-” refers to a deletion of one base pair. “a,” “g,” “c” and “t” in red and superscript refers to an insertion of an “A,” “G,” “C,” and “T,” respectively. **(E)** The *g1* mutant florets with (TG) or without transgene (TG-free) generated by AAC-G1.

We assembled another construct *AAC-G1* to target the *G1* gene, which takes part in suppressing the development of the sterile lemma. When *G1* is disrupted, the length of the sterile lemma increased dramatically, providing an easily scorable phenotype (Yoshida et al., 2009). We analyzed 88 T1 plants from 9 AAC-G1 T0 plants that displayed anthocyanin accumulation. The results of our visual screen for transgene-free plants (Figure 5A) were consistent with the results identified by PCR using primer pairs TG-F1/TG-R1 and TG-F2/TG-R2 (Figure 5B). For example, there were 5 and 2 transgene-free plants in the progeny of AAC-G1–#5 and AAC-G1–#6, respectively (Figures 5A,B). We also identified multiple alleles of *g1* (Figures 5C,D, Supplementary Table 2). It was clear that all of the T1 plants sequenced were either homozygous or bi-allelic (Supplementary Table 2). Our results demonstrated that the anthocyanin-based screening for transgene-free and edited plants were very effective (Figures 5D,E).

## Discussion

**Figure 6:**
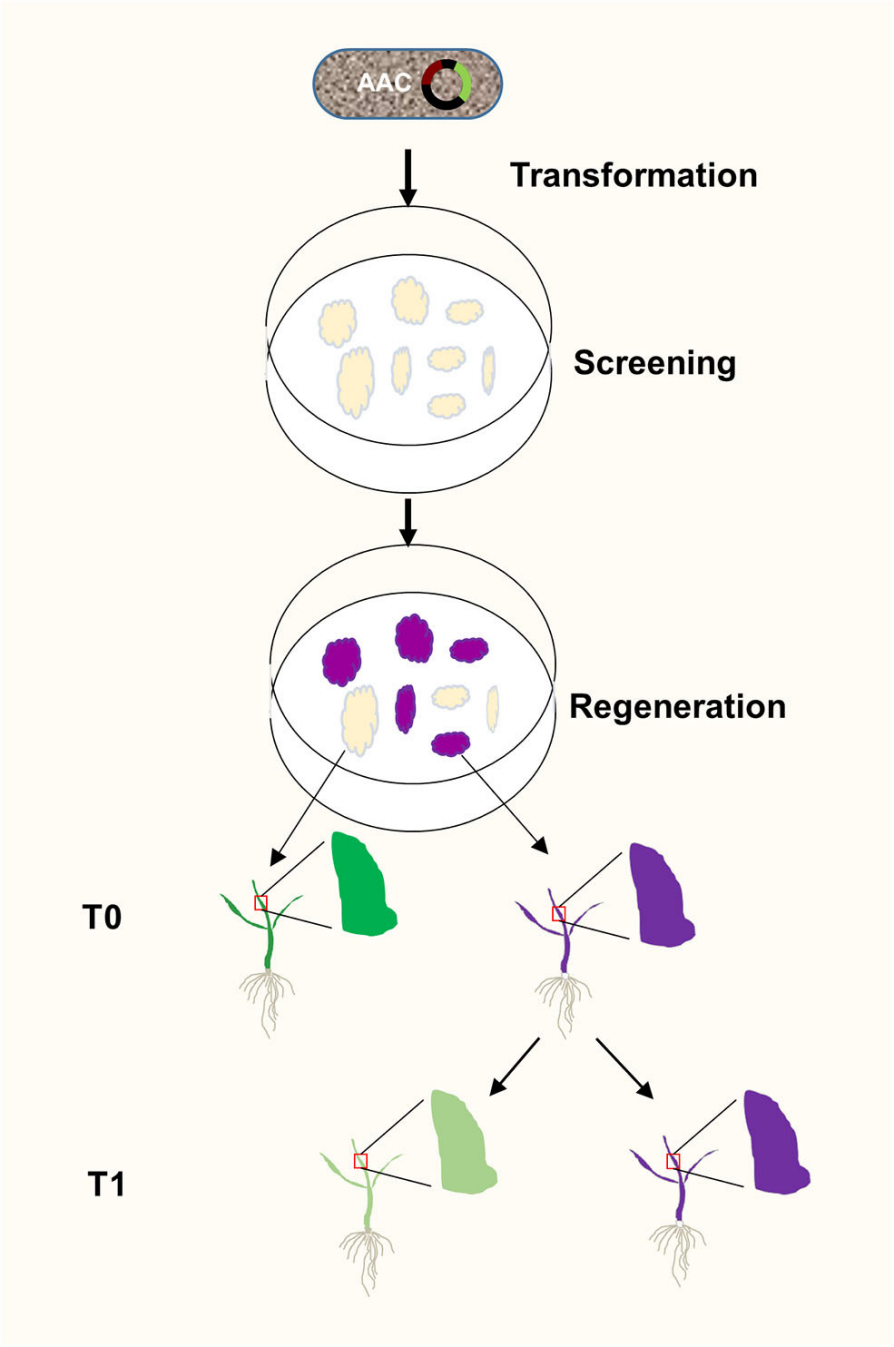
A flow chart of AAC-mediated isolation of transgene-free and target gene-edited rice plants. The *AAC* plasmid was transformed into rice calli through *Agrobacterium*-mediated transformation. At the calli stage, the *OsC1* gene was expressed to generate purple callus, and the target gene is presumably being edited by Cas9/sgRNA complex. Consequently, at T1 stage, the transgene free plants without purple were selected from the progeny of the purple T0 plants.

Plant transformation through tissue culture usually takes weeks before a positive result is obtained. The ability to identify transgenic events at the earliest stages of plant transformation offers advantages. We showed that anthocyanin biosynthesis can be activated at calli stage, providing a visual and functional assay for positive transformants very early on (Figure 6). Anthocyanin biosynthesis does not require exogenous substrates. It allows continuous monitoring transgene activities under sterile conditions, which is very useful in tissue culture. Because anthocyanin is a plant pigment existent in many plants including some rice varieties, it is not toxic to plants and causes fewer environmental impacts compared to antibiotic and herbicide-resistance screening. In addition, anthocyanins can absorb excessive ultraviolet lights and visible lights and remove free radicals to protect plants from ultraviolet rays, thereby providing protection for plants (Guo et al., 2008). Anthocyanins can also enhance plant resistance to drought and low temperature, and strengthen the ability to resist pathogenic bacteria, thereby protecting plants from biotic and abiotic stresses (Ahmed et al., 2014), Moreover, anthocyanins can also respond to external trauma by preventing oxidation (Gould et al., 2002), Therefore, the T0 plants generated using our AAC system might have better chance to survive after transferring them from the sterile environment to natural field.

We used the rice *ACTIN* promoter, which is a strong and ubiquitously active promoter, to drive *OsC1* expression. Interestingly, anthocyanin accumulation was not ubiquitously distributed (Figure 1). We observed obvious purple color in calli, the vascular tissue of the stems and leaves, and the grains. But some other tissues did not display the purple color (Figure 1B). Previous studies showed that the anthocyanin biosynthesis pathway is regulated by the MYB-bHLH-WD40 (MBW) complex (Xu et al., 2015; Zhu et al., 2017; Zheng et al., 2019), and MYB protein is the key component (Jaakola, 2013; Xu et al., 2015). Our activation of anthocyanin biosynthesis by expressing the transcription factor OsC1 only works in plants that have all functional anthocyanin biosynthetic genes and sufficient amount of bHLH and WD40. Many popular white rice varieties have defects in some of the anthocyanin genes (Zheng et al., 2019), rendering them not suitable for our AAC technology. Recently, we developed a novel color reporter RUBY (He et al., 2020), which can generate red color in all eukaryotic cells include rice. For those rice cultivars that AAC is not suitable, RUBY can be used for color-assisted CRISPR technology. We introduced the *pACTIN-OsC1* cassette to the rice variety Chao2-10 (Li et al., 2013b), which was previously shown to have all functional anthocyanin biosynthetic genes (Li et al., 2013b, 2017; Zheng et al., 2019), but lacked the *MYB* gene in the MBW complex. From a practical point view, the non-ubiquitous accumulation of anthocyanin actually is better because of its minimal impact on plant growth and development.

In our previous studies, we used antibiotics to screen the candidate gene-edited plants and found that some plants regenerated from antibiotic-resistance calli did not have target site mutations (He et al., 2017b). In this study, we found that all of the purple color plants were transgenic plants (Figures 2, 3, 5A,B) and all of the purple color plants had mutation at the target sites (Table 1, Supplementary Tables 1, 2). Our interpretation is that anthocyanin accumulation may be indicative of elevated Cas9 expression, which directly determines gene editing efficiency.

In summary, we have repurposed the anthocyanin biosynthesis pathway for serving as a visible marker for selecting plant transformation events and as a proxy for the presence of transgenes (Figure 6). Our system offers an alternative to fluorescence markers and antibiotic selections. It is especially powerful when used in tissue culture and in combination with gene editing machinery. The extend of anthocyanin accumulation might be indicative of the expression levels of other linked transgenes such as *Cas9*, thus providing a robust and speedy method for identifying plants with elevated gene editing efficiency.

## Materials and Methods

### The AAC Plasmid Construction

Primers used in this study were listed in the Supplementary Table 3. Our *AAC* plasmid contained two main expression cassettes: *OsACTIN1 promoter-OsC1* and *UBIQUITIN promoter-Cas9*. *OsACTIN1* promoter was amplified using primers of pCXUN-Act1PF and pCXUN-Act1PR from the plasmid *pCB2006*, which was kindly provided by Professor Lizhong Xiong (Xiao et al., 2009). The PCR product was cloned into *pCXUN-Cas9* (He et al., 2017b) at the *Kpn* I site. The *OsC1* gene was amplified from the cDNA plasmid provided by Dr. Hao Chen and Dr. Jie Zheng (Huazhong Agricultural University, Wuhan, China) by using primers of C1-Act1P-PCA9F and C1-Act1P-PCA9R, and the PCR fragment was inserted into the *Kpn* I site behind the *OsACTIN1* promoter to complete the *AAC* (Anthocyanin-marker Assisted CRISPR) plasmid construction. Guide RNA production cassette was inserted into the *Pme* I site of the *AAC* plasmid. We generated the plasmid *AAC-LA* and *AAC-G1*, which produce guide RNAs from the rice *U6* and *U3* promoter to target the rice *LAZY1* and *G1* gene by overlapping PCR, respectively (He et al., 2019). The correct clones were confirmed by sequencing with primer AAC-PmeI-seqF.

### Plant Transformation

The *AAC-LA* and *AAC-G1* plasmids were transformed into Chao2-10 through *Agrobacterium*-mediated plant transformation following a protocol that was previously described (Hiei et al., 1994). T0 plants were visually scored for color phenotype at calli culture stage and at different growing stages in natural field. Seeds from each individual T0 plants containing purple color in vascular tissue of stems and leaves were harvested separately.

### Characterization of T1 Plants

We randomly selected the T1 progenies of 11 T0 plants of AAC-LA and 9 independent AAC-G1 T0 plants to determine the efficiency of AAC system in editing the target genes. We used two primer pairs to amplify the specific regions of the AAC plasmids. TG-F1/TG-R1 was used to detect the presence of the *OsC1* expression cassette, and TG-F2/TG-R2 was used to amply part of the *CAS9* expression cassette. To check the quality of our genomic DNA samples, we used RG-F/RG-R for PCR reactions.

We used primer pairs LAZY1-GTF/LAZY1-GTF and G1-GTF/G1-GTR to amplify part of the *LAZY1* and *G1* genes from the AAC-LA and AAC-G1 T1 plants, respectively. We also directly sequenced the PCR products by using primers LAZY1-seq and G1-seq, respectively. For heterozygous or bi-allelic plants, the overlapping peaks were resolved using the publicly available Dsdecode site (http://skl.scau.edu.cn/dsdecode/) (Xie et al., 2017).

## Data Availability Statement

The raw data supporting the conclusions of this article will be made available by the authors, without undue reservation.

## Author Contributions

YH and YZ conceived the idea and wrote the first draft of the manuscript. YH, MZ, and JW conducted most of the experiments. MZ, JW, LO, RW, HS, LY, LW, MX, and HZ contributed to manuscript revision. All authors contributed to the article and approved the submitted version.

## Conflict of Interest

The authors declare that the research was conducted in the absence of any commercial or financial relationships that could be construed as a potential conflict of interest. The handling editor declared a past co-authorship with several of the authors YH, MX, and YZ.
