# Supplementary material for: Repurposing of Anthocyanin Biosynthesis for Plant Transformation and Genome Editing
Source: Front Genome Ed. 2020 Dec 3;2:607982. doi: 10.3389/fgeed.2020.607982 (PMC8525376; doi:10.3389/fgeed.2020.607982)
Supplement: Supplementary file 1 [file Data_Sheet_1.ZIP › Submitted-Table S1-V2.docx]

**Table S1. Mutations and segregation patterns in the T1 plants generated from the AAC-LA.**

| **Plants No.** | **Genotypes** | **Sequence analysis** | **Segregation** |
| --- | --- | --- | --- |
| WT | WT | AGGTGCTCGGCGGCGTCGACGTGGCGTCG**GCGCGGGTGAGCTTCGCCATGGG**CGGTGGCGTCGACTGCCCGCTCC |  |
|  |  |  |  |
| AAC-LA-#1 | HO, -45 | AGGTGCTCGG---------------------------------------------TGGCGTCGACTGCCCGCTCC  AGGTGCTCGG---------------------------------------------TGGCGTCGACTGCCCGCTCC | 6/8(1/6) |
|  | HO, -4 | AGGTGCTCGGCGGCGTCGACGTGGCGTCGGCGCGGGTGAGCT----CAT**GGG**CGGTGGCGTCGACTGCCCGCTCC  AGGTGCTCGGCGGCGTCGACGTGGCGTCGGCGCGGGTGAGCT----CAT**GGG**CGGTGGCGTCGACTGCCCGCTCC | 2/8(1/2) |
|  |  |  |  |
| AAC-LA-#2 | BI, -C/-10 | AGGTGCTCGGCGGCGTCGACGTGGCGTCGGCGCGGGTGAG----------GGCGGTGGCGTCGACTGCCCGCTCC  AGGTGCTCGGCGGCGTCGACGTGGCGTCGGCGCGGGTGAGCTTCGC-AT**GGG**CGGTGGCGTCGACTGCCCGCTCC | 18/40(3/18) |
|  | HO, -C | AGGTGCTCGGCGGCGTCGACGTGGCGTCGGCGCGGGTGAGCTTCGC-AT**GGG**CGGTGGCGTCGACTGCCCGCTCC  AGGTGCTCGGCGGCGTCGACGTGGCGTCGGCGCGGGTGAGCTTCGC-AT**GGG**CGGTGGCGTCGACTGCCCGCTCC | 12/40(2/12) |
|  | HO, -10 | AGGTGCTCGGCGGCGTCGACGTGGCGTCGGCGCGGGTGAG----------GGCGGTGGCGTCGACTGCCCGCTCC  AGGTGCTCGGCGGCGTCGACGTGGCGTCGGCGCGGGTGAG----------GGCGGTGGCGTCGACTGCCCGCTCC | 7/40(2/7) |
|  | BI, -40/-10 | AGGTGCTCGGCGGCGTCGACGTGGCGTCGGCGCGGGTGAG----------GGCGGTGGCGTCGACTGCCCGCTCC  AGGTGCTCGGCGGCGTCGACGTGGCGTC----------------------------------------CCGCTCC | 1/40(0/1) |
|  | BI, -19/-10 | AGGTGCTCGGCGGCGTCGACGTGGCGTCGGCGCGGGTG-------------------GCGTCGACTGCCCGCTCC  AGGTGCTCGGCGGCGTCGACGTGGCGTCGGCGCGGGTGAG----------GGCGGTGGCGTCGACTGCCCGCTCC | 1/40(1/1) |
|  | HE, -C/WT | AGGTGCTCGGCGGCGTCGACGTGGCGTCGGCGCGGGTGAGCTTCGC-AT**GGG**CGGTGGCGTCGACTGCCCGCTCC  AGGTGCTCGGCGGCGTCGACGTGGCGTCG**GCGCGGGTGAGCTTCGCCATGGG**CGGTGGCGTCGACTGCCCGCTCC | 1/40(1/1) |
|  |  |  |  |
| AAC-LA-#3 | HO, -411 | ACTACTTCTCCGGCGAC--(39bp)--CTCTTCACCTT--(372bp)--CGGTGGCGTCGACTGCC  ACTACTTCTCCGGCGAC--(39bp)--CTCTTCACCTT--(372bp)--CGGTGGCGTCGACTGCC | 4/9(0/4) |
|  | HO, -5 | AGGTGCTCGGCGGCGTCGACGTGGCGTCGGCGCGGGTGAG-----CCAT**GGG**CGGTGGCGTCGACTGCCCGCTCC  AGGTGCTCGGCGGCGTCGACGTGGCGTCGGCGCGGGTGAG-----CCAT**GGG**CGGTGGCGTCGACTGCCCGCTCC | 5/9(1/5) |
|  |  |  |  |
| AAC-LA-#4 | BI, -16/-71 | AGGTGCTCGGCGGCGTCGACGTGGCGTCGGCG----------------T**GGG**CGGTGGCGTCGACTGCCCGCTCC  AGGTGCTCGGCGGCGTCGACGTGGCGTCGGCGCGGGTGAG--(71bp)--AGTCGCGCCCGGAGTATCTGCAGGC | 15/26(5/15) |
|  | HO, -16 | AGGTGCTCGGCGGCGTCGACGTGGCGTCGGCG----------------T**GGG**CGGTGGCGTCGACTGCCCGCTCC  AGGTGCTCGGCGGCGTCGACGTGGCGTCGGCG----------------T**GGG**CGGTGGCGTCGACTGCCCGCTCC | 3/26(1/3) |
|  | HO, -71 | AGGTGCTCGGCGGCGTCGACGTGGCGTCGGCGCGGGTGAG--(71bp)--AGTCGCGCCCGGAGTATCTGCAGGC  AGGTGCTCGGCGGCGTCGACGTGGCGTCGGCGCGGGTGAG--(71bp)--AGTCGCGCCCGGAGTATCTGCAGGC | 8/26(5/8) |
|  |  |  |  |
| AAC-LA-#5 | BI, -3/-6 | AGGTGCTCGGCGGCGTCGACGTGGCGTCGGCGCGGGTGAGC------AT**GGG**CGGTGGCGTCGACTGCCCGCTCC  AGGTGCTCGGCGGCGTCGACGTGGCGTCGGCGCGGGTGAGCTTC---AT**GGG**CGGTGGCGTCGACTGCCCGCTCC | 3/7(1/3) |
|  | HO, -3 | AGGTGCTCGGCGGCGTCGACGTGGCGTCGGCGCGGGTGAGCTT---CAT**GGG**CGGTGGCGTCGACTGCCCGCTCC  AGGTGCTCGGCGGCGTCGACGTGGCGTCGGCGCGGGTGAGCTT---CAT**GGG**CGGTGGCGTCGACTGCCCGCTCC | 2/7(0/2) |
|  | HO, -6 | AGGTGCTCGGCGGCGTCGACGTGGCGTCGGCGCGGGTGAGC------AT**GGG**CGGTGGCGTCGACTGCCCGCTCC  AGGTGCTCGGCGGCGTCGACGTGGCGTCGGCGCGGGTGAGC------AT**GGG**CGGTGGCGTCGACTGCCCGCTCC | 2/7(0/2) |
|  |  |  |  |
| AAC-LA-#6 | BI, -32,S3/-42 | AGGTGCTCGGCG------------------------------------------GTGGCGTCGACTGCCCGCTCC  AGGTGCTCGGCGCCCTGGA--------------------------------GCGGTGGCGTCGACTGCCCGCTCC | 11/23(4/11) |
|  | HO, -32,S3 | AGGTGCTCGGCGCCCTGGA--------------------------------GCGGTGGCGTCGACTGCCCGCTCC  AGGTGCTCGGCGCCCTGGA--------------------------------GCGGTGGCGTCGACTGCCCGCTCC | 7/23(3/7) |
|  | HO, -42 | AGGTGCTCGGCG------------------------------------------GTGGCGTCGACTGCCCGCTCC  AGGTGCTCGGCG------------------------------------------GTGGCGTCGACTGCCCGCTCC | 5/23(2/5) |
|  |  |  |  |
| AAC-LA-#7 | BI, -32,S3/-42 | AGGTGCTCGGCG------------------------------------------GTGGCGTCGACTGCCCGCTCC  AGGTGCTCGGCGCCCTGGA--------------------------------GCGGTGGCGTCGACTGCCCGCTCC | 13/25(5/13) |
|  | HO, -32,S3 | AGGTGCTCGGCGCCCTGGA--------------------------------GCGGTGGCGTCGACTGCCCGCTCC  AGGTGCTCGGCGCCCTGGA--------------------------------GCGGTGGCGTCGACTGCCCGCTCC | 5/25(1/5) |
|  | HO, -42 | AGGTGCTCGGCG------------------------------------------GTGGCGTCGACTGCCCGCTCC  AGGTGCTCGGCG------------------------------------------GTGGCGTCGACTGCCCGCTCC | 7/25(1/7) |
|  |  |  |  |
| AAC-LA-#8 | BI, -6/-C | AGGTGCTCGGCGGCGTCGACGTGGCGTCGGCGCGGGTGAG------CAT**GGG**CGGTGGCGTCGACTGCCCGCTCC  AGGTGCTCGGCGGCGTCGACGTGGCGTCGGCGCGGGTGAGCTTCG-CAT**GGG**CGGTGGCGTCGACTGCCCGCTCC | 3/5(1/3) |
|  | HO, -6 | AGGTGCTCGGCGGCGTCGACGTGGCGTCGGCGCGGGTGAG------CAT**GGG**CGGTGGCGTCGACTGCCCGCTCC  AGGTGCTCGGCGGCGTCGACGTGGCGTCGGCGCGGGTGAG------CAT**GGG**CGGTGGCGTCGACTGCCCGCTCC | 1/5(0/1) |
|  | HO, -C | AGGTGCTCGGCGGCGTCGACGTGGCGTCGGCGCGGGTGAGCTTCG-CAT**GGG**CGGTGGCGTCGACTGCCCGCTCC  AGGTGCTCGGCGGCGTCGACGTGGCGTCGGCGCGGGTGAGCTTCG-CAT**GGG**CGGTGGCGTCGACTGCCCGCTCC | 1/5(0/1) |
|  |  |  |  |
| AAC-LA-#9 | BI, -22/+449 | AGGTGCTCGGCGGCGTCGAC----------------------TCGCCATGGGCGGTGGCGTCGACTGCCCGCTCC  TGGCGTCGGCGCGGGTGAGCTTCGC **replaced by**  CAATCGCCCACGTGGCCTGGCACCTTCGGGGACGTCGGGCCCGAGGGTGAGGTGTCCGCCCTCCTCCTGATTTCCCCCGAGGGGGGGGGGGTCGGGTTGCGCTTGCCCCGGCCCCGAGGGCCGAGGCACCCCGACCCCTTAAGGAAGTCTGCGCCACATATATGGGATAAGTGAGCACAGCTGTGCTCACCTAACAGCATTTATTGCAGTCTGGTCAAGCGTGTCACGCTGCATGCAACGCAGTACAGCGCGTTCCTTTATCCGGTCTGTGACCAGTCACAGACCAGTCAGATCACGGGTTAGGTGGCGACTGGCGGTCTGACGCACGCCTTGCCCCATCCCGTCAAGACGAAAGCCTCTAGGCTCTCGTCTCAAGCCGGAGCTAGCGTGTTATCTCTTAGAGATGGCACGTTAGCCCTGGTCAGATTTATACCAGGCTTCATCCTAACCATTACAGGCAAGGTGTTACACGAA | 5/13(1/5) |
|  | HO, -22 | AGGTGCTCGGCGGCGTCGAC----------------------TCGCCATGGGCGGTGGCGTCGACTGCCCGCTCC  AGGTGCTCGGCGGCGTCGAC----------------------TCGCCATGGGCGGTGGCGTCGACTGCCCGCTCC | 2/13(0/2) |
|  | HO, +449 | TGGCGTCGGCGCGGGTGAGCTTCGC **replaced by**  CAATCGCCCACGTGGCCTGGCACCTTCGGGGACGTCGGGCCCGAGGGTGAGGTGTCCGCCCTCCTCCTGATTTCCCCCGAGGGGGGGGGGGTCGGGTTGCGCTTGCCCCGGCCCCGAGGGCCGAGGCACCCCGACCCCTTAAGGAAGTCTGCGCCACATATATGGGATAAGTGAGCACAGCTGTGCTCACCTAACAGCATTTATTGCAGTCTGGTCAAGCGTGTCACGCTGCATGCAACGCAGTACAGCGCGTTCCTTTATCCGGTCTGTGACCAGTCACAGACCAGTCAGATCACGGGTTAGGTGGCGACTGGCGGTCTGACGCACGCCTTGCCCCATCCCGTCAAGACGAAAGCCTCTAGGCTCTCGTCTCAAGCCGGAGCTAGCGTGTTATCTCTTAGAGATGGCACGTTAGCCCTGGTCAGATTTATACCAGGCTTCATCCTAACCATTACAGGCAAGGTGTTACACGAA  TGGCGTCGGCGCGGGTGAGCTTCGC **replaced by**  CAATCGCCCACGTGGCCTGGCACCTTCGGGGACGTCGGGCCCGAGGGTGAGGTGTCCGCCCTCCTCCTGATTTCCCCCGAGGGGGGGGGGGTCGGGTTGCGCTTGCCCCGGCCCCGAGGGCCGAGGCACCCCGACCCCTTAAGGAAGTCTGCGCCACATATATGGGATAAGTGAGCACAGCTGTGCTCACCTAACAGCATTTATTGCAGTCTGGTCAAGCGTGTCACGCTGCATGCAACGCAGTACAGCGCGTTCCTTTATCCGGTCTGTGACCAGTCACAGACCAGTCAGATCACGGGTTAGGTGGCGACTGGCGGTCTGACGCACGCCTTGCCCCATCCCGTCAAGACGAAAGCCTCTAGGCTCTCGTCTCAAGCCGGAGCTAGCGTGTTATCTCTTAGAGATGGCACGTTAGCCCTGGTCAGATTTATACCAGGCTTCATCCTAACCATTACAGGCAAGGTGTTACACGAA | 6/13(2/6) |
|  |  |  |  |
| AAC-LA-#10 | BI, -3/-6 | AGGTGCTCGGCGGCGTCGACGTGGCGTCGGCGCGGGTGAGC------AT**GGG**CGGTGGCGTCGACTGCCCGCTCC  AGGTGCTCGGCGGCGTCGACGTGGCGTCGGCGCGGGTGAGCTTC---AT**GGG**CGGTGGCGTCGACTGCCCGCTCC | 4/8(1/4) |
|  | HO, -3 | AGGTGCTCGGCGGCGTCGACGTGGCGTCGGCGCGGGTGAGCTTC---AT**GGG**CGGTGGCGTCGACTGCCCGCTCC  AGGTGCTCGGCGGCGTCGACGTGGCGTCGGCGCGGGTGAGCTTC---AT**GGG**CGGTGGCGTCGACTGCCCGCTCC | 3/8(1/3) |
|  | HO, -6 | AGGTGCTCGGCGGCGTCGACGTGGCGTCGGCGCGGGTGAGC------AT**GGG**CGGTGGCGTCGACTGCCCGCTCC  AGGTGCTCGGCGGCGTCGACGTGGCGTCGGCGCGGGTGAGC------AT**GGG**CGGTGGCGTCGACTGCCCGCTCC | 1/8(0/1) |
|  |  |  |  |
| AAC-LA-#11 | BI, -29,S1/-43 | AGG-------------------------------------------CAT**GGG**CGGTGGCGTCGACTGCCCGCTCC  AGGTGCTCGGCGGCGTCGACGTGAC-----------------------------GTGGCGTCGACTGCCCGCTCC | 11/18(4/11) |
|  | HO, -29,S1 | AGGTGCTCGGCGGCGTCGACGTGAC-----------------------------GTGGCGTCGACTGCCCGCTCC  AGGTGCTCGGCGGCGTCGACGTGAC-----------------------------GTGGCGTCGACTGCCCGCTCC | 6/18(0/6) |
|  | HO, -43 | AGG-------------------------------------------CAT**GGG**CGGTGGCGTCGACTGCCCGCTCC  AGG-------------------------------------------CAT**GGG**CGGTGGCGTCGACTGCCCGCTCC | 1/18(0/1) |

“HO”, “HE”, “BI”, and “WT” represent homozygous, heterozygous, bi-allelic, and wild type genotypes, respectively. The numbers in the column of “Genotypes” mean the numbers of base pair changes in each line. The PAM site “GGG” required for Cas9 cleavage is marked in green. DNA sequence (genotype) of the T1 plants from individual T0 plants are as shown. “-” refers to a deletion of one base pair. “S3” and “S1” means three and one base pair(s) substitution, respectively. The last column shows the mutation segregation ratio, and the bracketed number refers to the ratio of transgene-free plants (without purple).
