# Supplementary material for: Repurposing of Anthocyanin Biosynthesis for Plant Transformation and Genome Editing
Source: Front Genome Ed. 2020 Dec 3;2:607982. doi: 10.3389/fgeed.2020.607982 (PMC8525376; doi:10.3389/fgeed.2020.607982)
Supplement: Supplementary file 1 [file Data_Sheet_1.ZIP › Submitted-Table S3.docx]

**Table S3. The primers used in this study**

| Primer | sequence |
| --- | --- |
| RG-F | CTCAACCCCAAGGCTAACAG |
| RG-R | ACCTCAGGGCATCGGAAC |
| TG-F1 | GTCGTTTCTTGACACCGACGAC |
| TG-R1 | GGTTTTTATGATTAGAGTCCCGCAA |
| TG-F2 | CCCTGCCTTCATACGCTATTT |
| TG-R2 | GACTTGAAGTTCGGGGTGAG |
| LAZY1-GTF | CCTGCAACTGCATCACCGGGCTTG |
| LAZY1-GTR | TCCAAGGAAACCTCATGAAATAGTCAGCCA |
| LAZY1-seq | GAAGCGGGTGCGCATGAACAGCTC |
| G1-GTF | GGCCAAGCCCACTAGACGCATTCTGT |
| G1-GTR | CGAAGCAACGGAACGAACACGTACGGA |
| G1-seq | GAGGACGCTCCCGAAGTCGCAGTA |
| pCXUN-Act1PF | TACGAATTCGAGCTCGGTACGCATACTCGAGGTCATTCATATGCTTGAG |
| pCXUN-Act1PR | ATCCCCCTTTCGCCAGGGGTACCGAGATCGTCGTCCGGCAGC |
| C1-Act1P-PCA9F | CCGGACGACGATCTCGGTACGGAGAATGGGGAGGAGAGCTTG |
| C1-Act1P-PCA9R | ATCCCCCTTTCGCCAGGGGTACCGTCACGCACACAAGTTCCAGGC |
| dPmeI-U6-F | GTCGTTTCCCGCCTTCAGTTTATGTACAGCATTACGTAGG |
| PmeI-U6-R | CTGTCAAACACTGATAGTTTAAACGATGGTGCTTACTGTTTAG |
| dPmeI-U3-F | GTCGTTTCCCGCCTTCAGTTTGTAATTCATCCAGGTCTCCAAG |
| PmeI-U3-R | CTGTCAAACACTGATAGTTTAAACGCTGTGCCGTACGACGGTACG |
| G1-U3_F | AACATGAACGGCGCCCGCGGGTTTTAGAGCTAGAAATAGCAAGTTA |
| G1-U3_R | CCGCGGGCGCCGTTCATGTTGCCACGGATCATCTGCACAACTC |
| LAZY1-U6_F | GCGCGGGTGAGCTTCGCCATGTTTTAGAGCTAGAAATAGCAAGTTA |
| LAZY1-U6_R | ATGGCGAAGCTCACCCGCGCAACCTGAGCCTCAGCGCAGC |
| AAC-PmeI-seqF | GAACGGATAAACCTTTTCACGCCC |
