# Supplementary material for: Repurposing of Anthocyanin Biosynthesis for Plant Transformation and Genome Editing
Source: Front Genome Ed. 2020 Dec 3;2:607982. doi: 10.3389/fgeed.2020.607982 (PMC8525376; doi:10.3389/fgeed.2020.607982)
Supplement: Supplementary file 1 [file Data_Sheet_1.ZIP › Submitted-Table S2-V2.docx]

**Table S2. Mutations and segregation patterns in the T1 plants generated from the AAC-G1.**

| **Plants No.** | **Genotypes** | **Sequence analysis** | **Segregation** |
| --- | --- | --- | --- |
| WT | WT | **CCGCCGCGGGCGCCGTTCATGTT** |  |
|  |  |  |  |
| AAC-G1-#1 | BI, +G/+A | **CCG**CCG^g^CGGGCGCCGTTCATGTT  **CCG**CCG^a^CGGGCGCCGTTCATGTT | 5/9(1/5) |
|  | HO, +A | **CCG**CCG^a^CGGGCGCCGTTCATGTT  **CCG**CCG^a^CGGGCGCCGTTCATGTT | 1/9(0/1) |
|  | HO, +G | **CCG**CCG^g^CGGGCGCCGTTCATGTT  **CCG**CCG^g^CGGGCGCCGTTCATGTT | 3/9(1/3) |
|  |  |  |  |
| AAC-G1-#2 | HO, -C | **CCG**CCG-GGGCGCCGTTCATGTT  **CCG**CCG-GGGCGCCGTTCATGTT | 8/8(2/8) |
|  |  |  |  |
| AAC-G1-#3 | BI, +T/-68 | **CCG**CCG^t^CGGGCGCCGTTCATGTT  ACCGCCACCG--(68)--TTCATGTTGC | 6/11(2/6) |
|  | HO, +T | **CCG**CCG^t^CGGGCGCCGTTCATGTT  **CCG**CCG^t^CGGGCGCCGTTCATGTT | 3/11(1/3) |
|  | HO, -68 | ACCGCCACCG--(68)--TTCATGTTGC  ACCGCCACCG--(68)--TTCATGTTGC | 2/11(0/2) |
|  |  |  |  |
| AAC-G1-#4 | BI, +A/-C | **CCG**CCG-GGGCGCCGTTCATGTT  **CCG**CCG^a^CGGGCGCCGTTCATGTT | 3/5(1/3) |
|  | HO, +A | **CCG**CCG^a^CGGGCGCCGTTCATGTT  **CCG**CCG^a^CGGGCGCCGTTCATGTT | 2/5(0/2) |
|  |  |  |  |
| AAC-G1-#5 | BI, +T/+A | **CCG**CCG^t^CGGGCGCCGTTCATGTT  **CCG**CCG^a^CGGGCGCCGTTCATGTT | 5/18(0/5) |
|  | HO, +A | **CCG**CCG^a^CGGGCGCCGTTCATGTT  **CCG**CCG^a^CGGGCGCCGTTCATGTT | 4/18(0/4) |
|  | HO, +T | **CCG**CCG^t^CGGGCGCCGTTCATGTT  **CCG**CCG^t^CGGGCGCCGTTCATGTT | 9/18(5/9) |
|  |  |  |  |
| AAC-G1-#6 | BI, -C/+C | **CCG**CCG-GGGCGCCGTTCATGTT  **CCG**CCG^c^CGGGCGCCGTTCATGTT | 10/15(1/10) |
|  | HO, +C | **CCG**CCG^c^CGGGCGCCGTTCATGTT  **CCG**CCG^c^CGGGCGCCGTTCATGTT | 3/15(1/3) |
|  | HO, -C | **CCG**CCG-GGGCGCCGTTCATGTT  **CCG**CCG-GGGCGCCGTTCATGTT | 2/15(0/2) |
|  |  |  |  |
| AAC-G1-#7 | BI, -C/+T | **CCG**CCG-GGGCGCCGTTCATGTT  **CCG**CCG^t^CGGGCGCCGTTCATGTT | 4/10(0/4) |
|  | HO, +T | **CCG**CCG^t^CGGGCGCCGTTCATGTT  **CCG**CCG^t^CGGGCGCCGTTCATGTT | 5/10(1/5) |
|  | HO, -C | **CCG**CCG-GGGCGCCGTTCATGTT  **CCG**CCG-GGGCGCCGTTCATGTT | 1/10(0/1) |
|  |  |  |  |
| AAC-G1-#8 | BI, +T/-C | **CCG**CCG-GGGCGCCGTTCATGTT  **CCG**CCG^t^CGGGCGCCGTTCATGTT | 5/8(0/5) |
|  | HO, +T | **CCG**CCG^t^CGGGCGCCGTTCATGTT  **CCG**CCG^t^CGGGCGCCGTTCATGTT | 2/8(0/2) |
|  | HO, -C | **CCG**CCG-GGGCGCCGTTCATGTT  **CCG**CCG-GGGCGCCGTTCATGTT | 1/8(1/1) |
|  |  |  |  |
| AAC-G1-#9 | HO, +A | **CCG**CCG^a^CGGGCGCCGTTCATGTT  **CCG**CCG^a^CGGGCGCCGTTCATGTT | 4/4(1/4) |

“HO”, “BI”, and “WT” represent homozygous, bi-allelic, and wild type genotypes, respectively. The numbers in the column of “Genotypes” mean the numbers of base pair changes in each line. The PAM site “CCG” required for Cas9 cleavage is marked in green. DNA sequence (genotype) of the T1 plants from individual T0 plants are as shown. “-” refers to a deletion of one base pair. “a”, “g”, “c” and “t” in red and superscript refers to an insertion of an “A”, “G”, “C” and “T”, respectively. The last column shows the mutation segregation ratio, and the bracketed number refers to the ratio of transgene-free plants (without purple).
